# Deep Learning Convolutional Neural Networks for the Automatic Quantification of Muscle Fat Infiltration Following Whiplash Injury

**DOI:** 10.1038/s41598-019-44416-8

**Published:** 2019-05-28

**Authors:** Kenneth A. Weber, Andrew C. Smith, Marie Wasielewski, Kamran Eghtesad, Pranav A. Upadhyayula, Max Wintermark, Trevor J. Hastie, Todd B. Parrish, Sean Mackey, James M. Elliott

**Affiliations:** 1 Systems Neuroscience and Pain Lab, Department of Anesthesiology, Perioperative and Pain Medicine, Stanford University, Palo Alto, CA USA; 2 School of Physical Therapy, Regis University, Denver, CO USA; 3 Department of Physical Therapy and Human Movement Sciences, Feinberg School of Medicine, Northwestern University, Chicago, IL USA; 4 Department of Radiology, Neuroradiology Section, Stanford University, Palo Alto, CA USA; 5 Statistics Department, Stanford University, Palo Alto, CA USA; 6 Department of Radiology, Northwestern University, Chicago, IL USA; 7 Honorary Senior Fellow, School of Health and Rehabilitation Sciences, The University of Queensland, Queensland, Australia; 8 Northern Sydney Local Health District, The Kolling Research Institute and The Faculty of Health Sciences, The University of Sydney, St. Leonards, NSW Australia

**Keywords:** Skeletal muscle, Prognostic markers

## Abstract

Muscle fat infiltration (MFI) of the deep cervical spine extensors has been observed in cervical spine conditions using time-consuming and rater-dependent manual techniques. Deep learning convolutional neural network (CNN) models have demonstrated state-of-the-art performance in segmentation tasks. Here, we train and test a CNN for muscle segmentation and automatic MFI calculation using high-resolution fat-water images from 39 participants (26 female, average = 31.7 ± 9.3 years) 3 months post whiplash injury. First, we demonstrate high test reliability and accuracy of the CNN compared to manual segmentation. Then we explore the relationships between CNN muscle volume, CNN MFI, and clinical measures of pain and neck-related disability. Across all participants, we demonstrate that CNN muscle volume was negatively correlated to pain (R = −0.415, p = 0.006) and disability (R = −0.286, p = 0.045), while CNN MFI tended to be positively correlated to disability (R = 0.214, p = 0.105). Additionally, CNN MFI was higher in participants with persisting pain and disability (p = 0.049). Overall, CNN’s may improve the efficiency and objectivity of muscle measures allowing for the quantitative monitoring of muscle properties in disorders of and beyond the cervical spine.

## Introduction

Muscle fat infiltration (MFI) has been described by conventional (T_1_- and T_2_-weighted) and advanced (Dixon and proton density fat fraction) magnetic resonance imaging (MRI) in cervical spine conditions, such as degenerative cervical myelopathy (DCM)^1,2^, spinal cord injury (SCI)^3,4^, and whiplash from a motor vehicle collision (MVC)^5–8^. While the mechanisms underlying these conditions greatly differ, the patterns of MFI appear consistent with the greatest magnitude occurring in the deepest, and most architecturally complex, muscular layer of the cervical extensors (i.e., multifidus and semispinalis cervicis)^5–7^.

While plausible to hypothesize that larger amounts of paraspinal MFI negatively impact function, recent studies do not provide confirmation^9,10^. This could be due to the varied methods used to measure MFI and function^11^, as others have shown MFI to be associated with lower physical function^12–14^, and that better surgical outcomes are achieved in those with larger muscle size and better quality^15,16^. Preliminary evidence suggests MFI may be reversible and associated with a concomitant improvement in chronic whiplash-related disability^17^.

Muscular degeneration (as the larger magnitude of MFI might indicate) may have clinical implications for management and rates of recovery from persistent spinal disorders that currently feature high as the world’s most disabling diseases: low back pain (first) and neck pain (fourth)^18^. Despite ever-increasing options for ‘treatment’ of these conditions, explanations for their persistence have become urgently needed^19^. MFI may be one neurobiological explanation^11,20,21^. However, manual segmentation methods for MFI do not permit for time-efficient monitoring of these muscles in clinical practice.

The recent application of deep learning methods (i.e., convolutional neural networks (CNN’s)) in medical imaging analysis has demonstrated impressive gains in image segmentation, with accuracy nearing human-level performance^22–24^. Accordingly, CNN’s may permit time-efficient quantification of the characteristic MFI observed in disorders of the spine (e.g., DCM, SCI, whiplash, and low back pain) and other musculoskeletal/neuromuscular conditions (e.g., rotator cuff pathology, osteoarthritis, diabetes, and laminopathies)^25^.

In this study, we trained and tested a CNN for segmentation of the deep cervical spine extensor muscles using high-resolution fat-water Dixon images from participants with whiplash following an MVC. We leveraged a previously developed CNN for the segmentation of medical images, V-Net, and the newly released deep learning platform, NiftyNet^26,27^. Then, we assessed the association of the automated CNN measures to clinical measures of pain and neck-related disability.

## Results

### CNN accuracy and reliability

Training the V-Net model was completed in 25,000 iterations. The trained CNN segmented every axial slice from the C3 to C7 vertebrae in less than 60 s per image. Accuracy of the trained CNN was evaluated on the testing dataset (n = 14). The average muscle volume ± 1 standard deviation (SD) for the CNN on the testing dataset was 34.9 ± 6.2 ml and 34.1 ± 6.1 ml for the left and right muscles, respectively, and the average MFI ± 1 SD was 20.7 ± 3.5% and 20.2 ± 4.3% for the left and right muscles, respectively.

**Figure 1:**
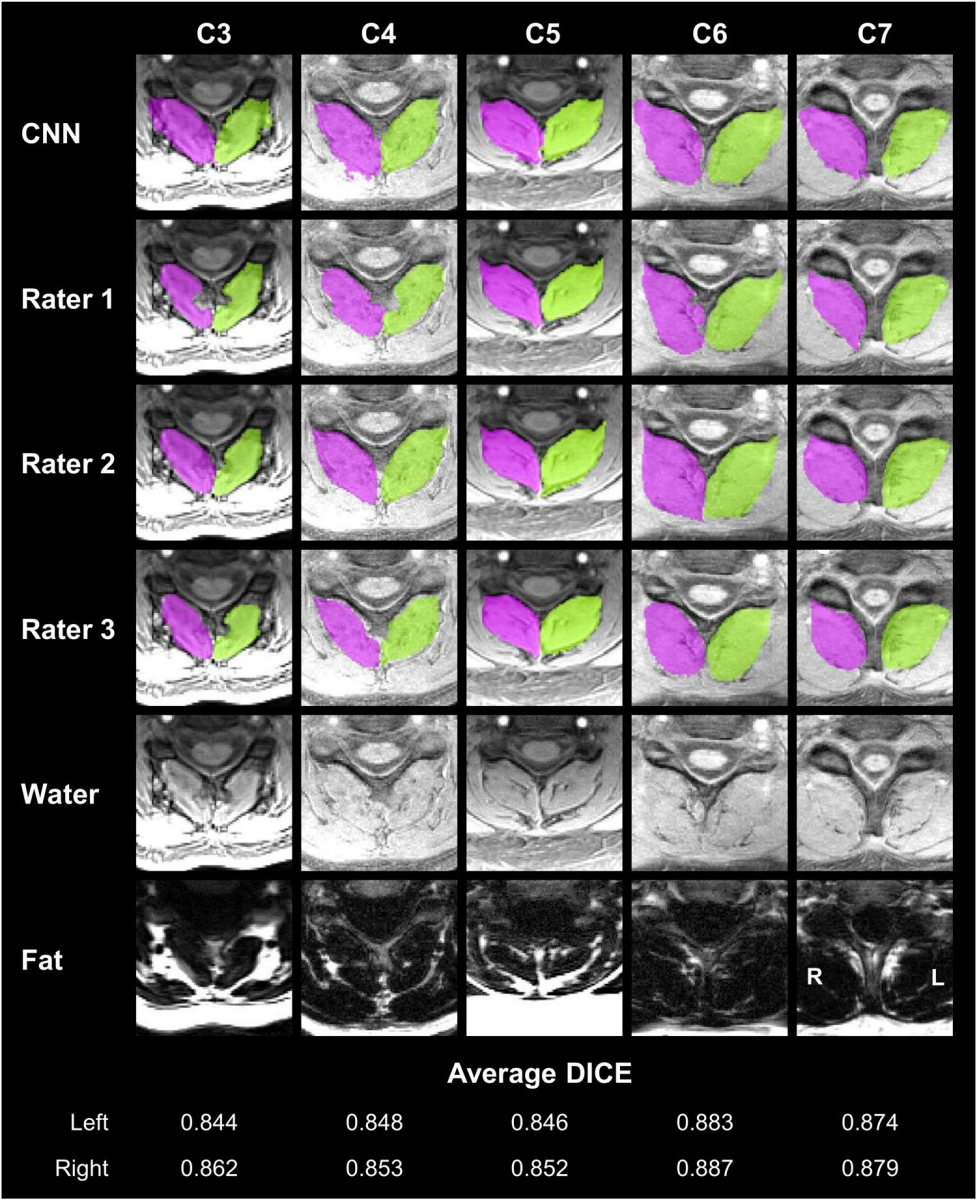
Convolutional neural network (CNN) segmentation results of the deep cervical extensors. CNN segmentation masks of the left (green) and right (magenta) deep cervical extensors (i.e., multifidus and semispinalis cervicis) are shown from five randomly selected testing datasets. Example axial images at the C3 to C7 vertebral levels were selected to show changes in the deep extensor muscle morphometry across the cervical spine. For comparison, the segmentation masks from each rater are also shown (rows 2–4). The bottom two rows show the water-only and fat-only images for reference. For each example, the average DICE between the CNN and each rater is reported for the left and right masks. The C3 vertebral level is from the inferior portion of the C3 vertebra. L = left, R = right.

Overall, we report high accuracy of the CNN on the testing dataset (Fig. 1). The average DICE ± 1 SD for the left and right deep cervical extensors was 0.862 ± 0.017 and 0.871 ± 0.016, respectively. The CNN model had high sensitivity (average true positive rate = 0.904 ± 0.021 and 0.908 ± 0.017 for the left and right muscles, respectively) and high precision (average positive predictive value = 0.829 ± 0.031 and 0.843 ± 0.032 for the left and right muscles, respectively). CNN segmentation performance was similar at the C3–C4 and C5–C6 vertebral levels where the deep cervical extensor muscle composition and morphometry differs. The average DICE was 0.876 ± 0.024 and 0.883 ± 0.026 at C3–C4 and 0.865 ± 0.020 and 0.866 ± 0.025 at C5–C6 for the left and right deep cervical extensors, respectively. The CNN performance metrics of the testing dataset are summarized in Table 1. Each rater’s masks were used as the ground truth (GT) to evaluate the performance of the CNN, and the average measures across the three raters were reported.

**Table 1:** Segmentation Performance Metrics of the CNN on the Testing Dataset (n = 14).

Performance Metric	Left	Right
Sørensen–Dice Index	0.862 ± 0.017	0.871 ± 0.016
Jaccard Index	0.758 ± 0.026	0.772 ± 0.026
Conformity Coefficient	0.678 ± 0.046	0.703 ± 0.044
True Positive Rate	0.904 ± 0.021	0.908 ± 0.017
True Negative Rate	0.999 ± < 0.001	0.999 ± < 0.001
Positive Predictive Value	0.829 ± 0.031	0.843 ± 0.032
Volume Ratio	1.100 ± 0.058	1.087 ± 0.058

Metrics shown = average ± 1 standard deviation.

**Figure 2:**
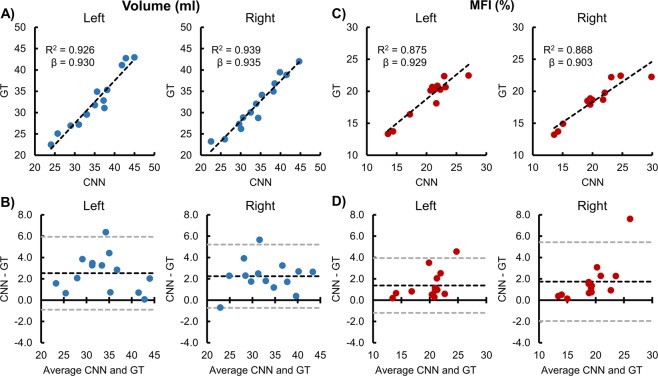
Reliability and accuracy of the convolutional neural network (CNN) segmentation on the testing dataset (n = 14). Correlation and Bland-Altman plots are shown for the left and right deep cervical extensor muscle volumes and muscle fat infiltration (MFI). The average measures of the three raters were used as the ground truth (GT). (**A**,**C**) The dashed black line represents the best fit line. The linear regression coefficient (β) of GT on CNN (intercept = 0) is also provided, which can be used to correct the CNN measurement bias. (**B**,**D**) The dashed black and gray lines indicate the average difference (i.e., bias) ± 1.96 × standard deviation (i.e., 95% limits of agreement).

Interrater reliability between the three raters was excellent with the intraclass correlation coefficients (ICC_2,1_) for the left volume, right volume, left MFI, and right MFI equal to 0.85, 0.83, 0.90, and 0.92, respectively. Using the average measures across the raters, the interrater reliability between the raters and the CNN model was also excellent with the ICC_2,1_ for the left volume, right volume, left MFI, and right MFI equal to 0.94, 0.95, 0.92, and 0.88, respectively. The average difference in volume (CNN − GT) was 2.5 ml (95% confidence interval (CI) 1.5–3.5) and 2.2 ml (1.4–3.1) for the left and right muscles, respectively, indicating that the CNN overestimated (i.e., bias) the muscle volume compared to the GT. Similarly, the average difference in MFI (CNN − GT) was 1.4% (95% CI 0.6–2.1) and 1.7% (0.6–2.8) for the left and right muscles, respectively, indicating that the CNN also demonstrated a bias towards higher MFI compared to the GT (Fig. 2).

### Association with clinical measures of pain and neck-related disability

**Figure 3:**
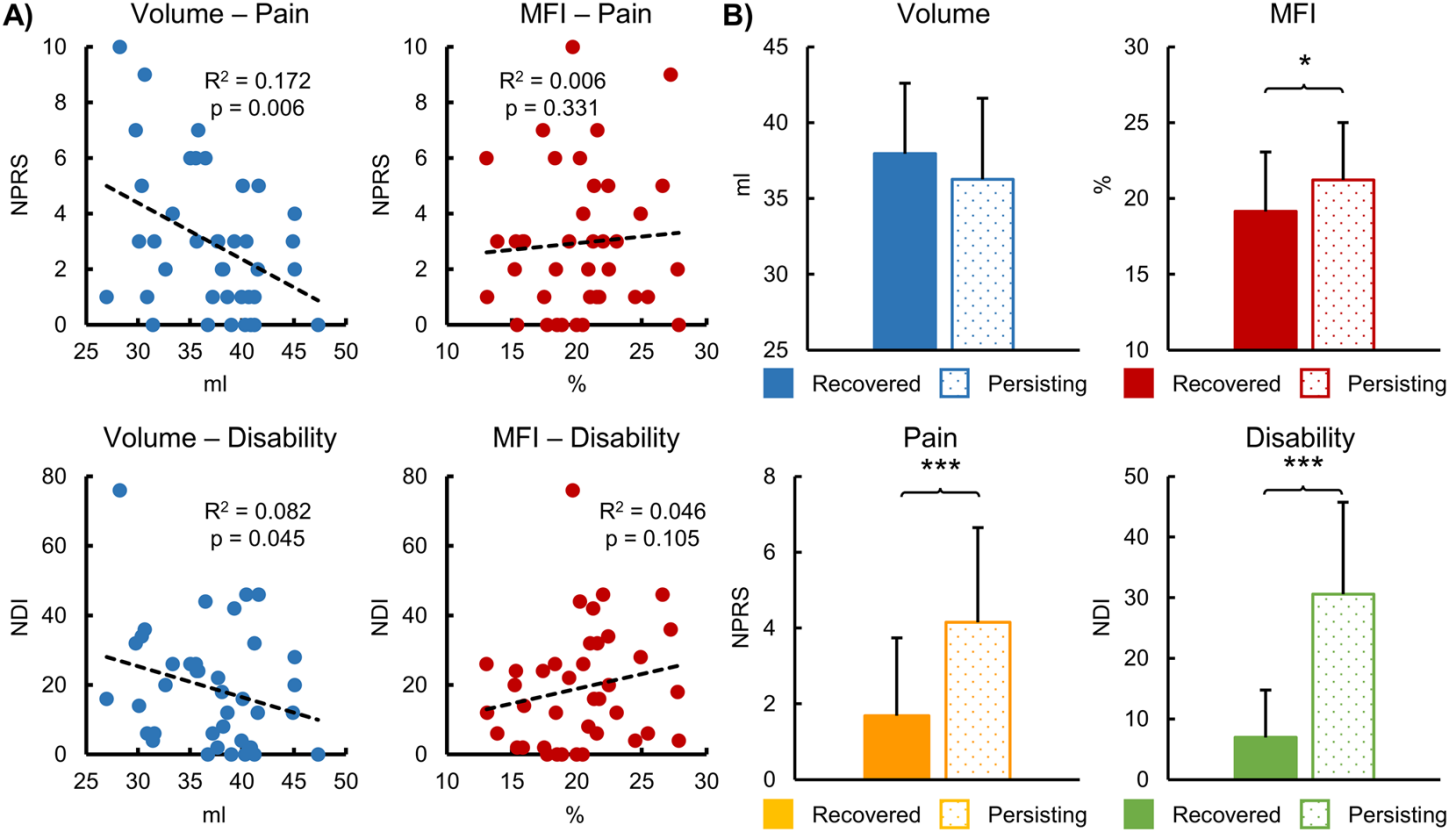
Associations between convolutional neural network (CNN) muscle volume and muscle fat infiltration (MFI) and the clinical measures of pain and neck-related disability. Pain and neck-related disability were assessed using the numerical pain rating scale (NPRS) and the neck disability index (NDI), respectively. (**A**) Muscle volume was significantly negatively correlated with both pain and neck-related disability. A non-significant positive correlation between MFI and neck-related disability was present but not between MFI and pain. (**B**) The dataset was then split into groups of recovered (NDI ≤ 28, n = 19) versus persisting (NDI > 28, n = 20) whiplash using the NDI at 3 months post motor vehicle collision. The persisting group had significantly higher pain and neck-related disability compared to the recovered group. MFI was significantly higher in the persisting group compared to the recovered group. Muscle volume and MFI were corrected for age, gender, and body mass index. Error bars = 1 standard deviation. *p < 0.05, ***p < 0.001.

Muscle volume was significantly negatively correlated to pain (R = −0.415, p = 0.006) and neck-related disability (R = −0.286, p = 0.045). MFI tended to be positively correlated to neck-related disability (R = 0.214, p = 0.105) but not pain (R = 0.075, p = 0.331) (Fig. 3A). Average pain (t = −3.356, df = 37, p < 0.001) and neck-related disability (t = −6.060, df = 37, p < 0.001) were significantly higher in the persistent group (neck disability index (NDI) > 28, n = 20, 16 female, average age = 33.3 ± 9.5 years, body mass index (BMI) = 23.8 ± 3.2 kg/m^2^) compared to the recovered group (NDI ≤ 28, n = 19, 10 female, average age = 29.9 ± 9.1 years, BMI = 25.8 ± 4.0 kg/m^2^). As hypothesized, MFI (t = 1.696, df = 37, p = 0.049) was significantly higher in the persistent group compared to those in the recovered group. While muscle volume was lower in the persistent group compared to those in the recovered group, the difference was not significant (t = −1.036, df = 37, p = 0.154) (Fig. 3B). Pain, neck-related disability, muscle volume, and MFI for each group are summarized in Table 2.

**Table 2:** CNN Muscle and Clinical Measures for Recovered and Persisting Whiplash.

Measure	Recovered (n = 19)	Persisting (n = 20)	P-value
NPRS	1.7 ± 2.1	4.2 ± 2.5	< 0.001
NDI	6.9 ± 7.9	30.6 ± 15.2	< 0.001
Muscle Volume (ml)	37.9 ± 4.7	36.3 ± 5.3	0.154
MFI (%)	19.1 ± 3.9	21.2 ± 3.8	0.049

Recovery from whiplash was defined as an NDI ≤ 28 at 3 months post motor vehicle collision. NPRS = numerical pain rating scale, NDI = neck disability index. Metrics shown = average ± 1 standard deviation. Muscle volume and muscle fat infiltration (MFI) were corrected for age, gender, and body mass index. P-value based on one-tailed independent samples t-tests.

## Discussion

In this study, we trained and tested a CNN for segmentation of the deep cervical spine extensor muscles from high-resolution fat-water Dixon MRI datasets of participants with whiplash injury using V-Net and the NiftyNet deep learning platform. Overall, we demonstrate the feasibility of training a previous CNN model for a novel segmentation task. The trained CNN was highly efficient (< 60 s compared to ≈ 20 minutes for manual segmentation) in processing an image, and we report high accuracy and reliability of the CNN compared to manual segmentation for both the muscle volume and MFI measures. The association of the automated CNN measures to clinical measures of pain intensity and neck-related disability was also recognized. Lower muscle volume was associated with higher pain and higher neck-related disability, and higher MFI was present in participants with persisting whiplash versus those that recovered, supporting the validity and clinical utility of these measures.

Our findings are consistent with previous work from three different countries with different insurance schemes (Australia, United States, and Sweden), all demonstrating larger magnitudes of cervical spine extensor MFI in those with more severe levels of whiplash-related disability^5–7,28,29^. Participants nominating full recovery or mild symptoms do not present with the same magnitude of MFI. As such, the expression of MFI may embody one neurobiological basis underlying the transition to chronicity in a discrete, but not insignificant, number of these patients with persistent whiplash. It is noteworthy that the findings of MFI are not unique to whiplash injury, as similar MFI profiles have been observed, and reported, in DCM^1,2^, SCI^3^, but not idiopathic neck pain^8,30^, suggesting degenerative, and potentially traumatic, factors play a role in their development. More mechanistic work for understanding why and how MFI develops and its influence on recovery from trauma and other common degenerative processes is warranted and underway.

The CNN demonstrated a bias towards higher muscle volumes and MFI compared to manual segmentation. Continued training of the model, training on a larger and more diverse dataset, or the adjustment of the model hyperparameters may have reduced the bias and improved the accuracy. The increased muscle volume could also be due to an intrinsic property of the V-Net architecture, leading to dilation of the output, possibly as the information is compressed and decompressed through the convolutional and deconvolutional layers. The higher segmentation volume likely led to the inclusion of extramuscular fat located adjacent to the deep cervical extensors, which would contribute to the higher MFI measure. However, the average bias of the CNN was small and did not preclude us from identifying associations with the clinical measures. Because no specific clinical cutoffs or normative comparative datasets yet exist for these measures, we are not able to assess the clinical significance of the bias. More testing is necessary to fully understand the properties and behavior of the network to improve the accuracy and relationships to the clinical measures.

The NiftyNet platform supports multi-modal CNN models and 3D convolutional layers. In the present study, 2D convolutional layers were employed using the water-only axial images as features. The inclusion of the fat-only images and use of 3D convolutional layers may provide additional information to more accurately segment the muscles, but at the trade-off of a greater number of features, larger network size, higher model complexity, and increased computational costs. The reason for our choice was that our group mainly uses the axial water-only images for segmentation of the deep cervical extensors, and intuitively, we feel that they contain the most information^31^. We are actively exploring the inclusion of multi-modal features and different network architectures. The use of dilated convolutional layers in the multi-modal models may help reduce the feature space while maintaining spatial coverage and accuracy^23^.

The use of images from the same site, scanner, sequence, and imaging parameters reduces the generalizability of the trained model and is a recognized limitation. A major barrier in developing deep learning models for medical imaging tasks is the availability of large, diverse annotated datasets for model training. Fortunately, several collaborative efforts are currently in progress to pool clinically- and research-based imaging data towards the development of large multi-site (and multi-cultural) annotated imaging databases for research purposes. The use of multi-site imaging data will have its own inherit challenges that include, but are not limited to, developing/establishing analysis pipelines and models that can generalize to images of varying resolution, field-of-view, and orientation while also accounting for variability in image signal and contrast due to differences in the imaging parameters and equipment (TR, TE, imaging field strength, and manufacturer).

CNN models have potential to provide an efficient, accurate, and objective measure of muscle volume and MFI. Future directions will aim to refine CNN hyperparameters, compare different CNN models, explore the use of multi-modal imaging, obtain larger multi-site annotated imaging datasets to increase performance and generalizability, and establish a global resource of normative reference values where clinical comparisons can be informed on a patient-by-patient basis.

## Methods

### Participants

MRI datasets from 39 participants (26 female, average age ± 1 SD = 31.7 ± 9.3 years) were obtained from a prospective observational longitudinal study exploring recovery from whiplash (ClinicalTrials.gov Identifier: NCT02157038). Datasets from the third time point at 3-months post MVC were used in the present study. Inclusion criteria included age 18 to 65 years, Quebec Task Force whiplash grades of II to III, and < 1 week post MVC with a primary complaint of neck pain^32^. Exclusion criteria included spinal fracture from the MVC, history of a previous MVC, previous spinal surgery, previous diagnosis of cervical or lumbar radiculopathy, history of neurological or metabolic disorders, and standard contraindications to MRI. The study was approved by Northwestern University’s Institutional Review Board. All applicable institutional and governmental regulations concerning the ethical use of human volunteers were followed during the course of this research according to the Declaration of Helsinki, and written informed consent was obtained from every participant. Prior to working with the datasets, all personal identifying information was removed.

### Image acquisition and processing

Imaging was performed on a 3.0 T Siemens (Munich, Germany) Prisma scanner equipped with a 64-channel head/neck coil. High-resolution 3D fat-water images of the cervical and upper thoracic spine were acquired using a dual-echo gradient-echo FLASH sequence (2-point Dixon, TR = 7.05 ms, TE_1_ = 2.46 ms, TE_2_ = 3.69 ms, flip angle = 12°, bandwidth = 510 Hz/pixel, FOV = 190 × 320 mm^2^, slab oversampling of 20% with 40 partitions to prevent aliasing in the anterior-posterior direction, in-plane resolution = 0.7 × 0.7 mm^2^, slice thickness = 3.0 mm, number of averages = 6, acquisition time = 4 min 5 s)^33^. Fat and water have slightly different chemical structures and precessional frequencies that differentially influence the local magnetic field. Images can be acquired when the fat and water signals are in-phase (IP = W + F) and out-of-phase (OOP = W − F). The in-phase and out-of-phase images can be combined to create images with fat-only signal (F = (IP − OOP)/2) and water-only signal (W = (IP + OOP)/2). As the images are acquired simultaneously in the same sequence and space, the images require no registration. Three blinded, independent raters, each doctoral-level health professionals, with extensive training in the cervical spine anatomy and musculoskeletal imaging, manually segmented the left and right deep cervical extensor muscles (i.e., multifidus and semispinalis cervicis) from the water-only images using methods previously described (2018)^31^. The segmentation masks contained the background, left muscle group, and right muscle group, labeled as 0, 1, and 2, respectively.

### Data augmentation

Data augmentation is a step commonly used to supplement the size of the training dataset. The images were randomly split into training (n = 25) and testing (n = 14) datasets, and 5,100 augmented datasets were generated by applying a series of random affine spatial transformations (scaling, shearing, rotation, translation, and reflection) and adding varying degrees of Gaussian noise to a training image. For each augmented dataset, the same spatial transformations were applied to the segmentation mask from one randomly selected rater for use as the GT. A similar approach using each rater as the GT was used by Perone *et al*. (2018) for model training, forcing the model to learn the optimal weights for segmentation despite the interrater variability^23^. The augmented datasets were then split into final training (n = 5,000) and validation (n = 100) datasets.

### V-Net

V-Net is a CNN designed for segmentation tasks. The network consists of several stages having one or more convolutional layers (5 × 5 kernels with stride 1 and padding) followed by a PReLu activation function to extract features. The last step of each stage is a convolutional or de-convolutional layer (2 × 2 kernels with stride 2) to decrease or increase the resolution, respectively. The first half of the network contracts the resolution, while the second half expands the resolution back to the input dimensions. At each stage, a residual learning framework is implemented by adding the input of each stage to the output of its last convolutional layer. Fine feature information from each convolutional stage is also forwarded to the corresponding de-convolutional stage to improve the contour prediction. To limit bias towards predicting the image background, a loss function based on the Sørensen-Dice index (DICE) was employed and minimized. The output after soft-max transformation is probabilistic segmentation masks for the left and right muscles with the same dimensions as the input volume^26^.

### Training

V-Net was trained on the water-only images from the augmented training dataset using NiftyNet (Version 0.2.2, spatial window = 256 × 256, window orientation = axial, padding = 128 × 128, learning rate = 0.001, optimizer = Adam, loss function = DICE, decay = 0.0001, samples per volume = 30, window sampling = uniform, batch size = 30). NiftyNet is an open-source CNN platform built on TensorFlow (Version 1.7) in Python (Version 2.7) and designed specifically for medical imaging analysis^27^. Prior to training, histogram standardization was performed, the images were resampled to 0.5 × 0.5 × 0.5 mm^3^, mean-centered (i.e., mean subtracted from each image), and normalized by their standard deviation. The V-Net model was initialized with random weights, and training was completed once the average DICE plateaued on the validation dataset.

### Performance

Performance of the CNN was assessed using DICE, Jaccard index, conformity coefficient, true positive rate, true negative rate, positive predictive value, and volume ratio (Table 3)^34^. Muscle volume and MFI were measured for the left and right deep cervical extensors using the segmentation masks from each rater and the CNN model. MFI was calculated as the average fat-only signal divided by the sum of the average fat-only signal and the average water-only signal multiplied by 100. Reliability between the raters and the CNN was assessed using intraclass correlation coefficients (ICC_2,1_), Pearson correlations, and Bland-Altman plots.

**Table 3:** Segmentation Performance Metrics.

Metric	Equation	Range	Meaning
Sørensen-Dice Index (DICE)	$\frac{2\times |SM\cap GT|}{|SM|+|GT|}$	0–1	Spatial overlap between masks
Jaccard Index	$\frac{|SM\cap GT|}{|SM|+|GT|-|SM\cap GT|}$	0–1	Spatial overlap between masks
Conformity Coefficient	$1-\frac{FP+FN}{TP}$	≤ 1	Ratio of incorrectly and correctly segmented voxels
True Positive Rate (TPR)	$\frac{TP}{TP+FN}$	0–1	Sensitivity
True Negative Rate (TNR)	$\frac{TN}{TN+FP}$	0–1	Specificity
Positive Predictive Value (PPV)	$\frac{TP}{TP+FP}$	0–1	Precision
Volume Ratio	$\frac{|SM|}{|GT|}$	≥ 0	Ratio of mask volumes

SM = segmentation mask; GT = ground truth mask; TP = true positive, voxels correctly segmented as deep cervical extensor muscle; TN = true negative, voxels correctly segmented as background; FP = false positive, voxels incorrectly segmented as deep cervical extensor muscle; FN = false negative, voxels incorrectly segmented as background. The masks from each of the three raters were used as the GT for the performance metrics.

### Clinical measures

To investigate the association of the CNN muscle volume and MFI and the clinical measures, the complete dataset (n = 39) was then input into the trained CNN, and segmentation masks were generated. The left and right muscle volume and MFI of the deep cervical extensors were then calculated and averaged. For pain, the 11-point numerical pain rating scale (NPRS) with anchors of “no pain” (0) and “extreme pain” (10) was used to assess neck pain^35^. Neck-related function was assessed with the NDI^36^. The NDI is a 10-item scaled questionnaire that assesses disability and functioning specific to the neck. The scores range from 0 to 100, and higher values indicate more neck-related disability in daily activities.

Relationships between muscle volume and MFI, and the clinical measures of pain and neck-related disability, were assessed with partial Pearson correlations correcting for age, gender, and BMI. We hypothesized that lower muscle volume and higher MFI would be correlated with higher pain and disability. Next, we divided the dataset into groups of recovered (NDI ≤ 28) versus persisting whiplash (NDI > 28) using the NDI at 3 months post MVC. Differences in average muscle volume and MFI between the recovered and persistent groups were assessed with independent samples t-tests after correcting for age, gender, and BMI. We hypothesized that the group reporting persistent disability and higher pain intensity would have lower muscle volume and increased MFI compared to the recovered group. As the hypotheses were directional, one-tailed tests were performed with an α < 0.05 considered statistically significant. Statistical analyses were performed using IBM SPSS Statistics (Version 21, Armonk, NY, USA).

## Data Availability

The de-identified datasets used in this study are available from the corresponding author upon reasonable request.
